# Heavy Study Investment: An Analysis of the Defense Mechanisms Characterizing Studyholism and Study Engagement

**DOI:** 10.3390/ijerph19159413

**Published:** 2022-08-01

**Authors:** Yura Loscalzo, Marco Giannini

**Affiliations:** Department of Health Sciences, School of Psychology, University of Florence, 50135 Florence, Italy; marco.giannini@unifi.it

**Keywords:** study addiction, workaholism, work addiction, heavy work investment, work engagement, OCD, internalizing, obsessive, compulsion, study

## Abstract

Defense mechanisms are unconscious processes that protect a person from excessive anxiety. They are part of everyday functioning, and mature defenses are associated with positive outcomes. However, the excessive use of defenses or the use of immature defenses is associated with psychopathology. The present study aims to analyze the defense mechanisms that characterize two types of heavy study investment: Studyholism and Study Engagement. We performed a path analysis, MANOVAs, and binary logistic regressions on 422 Italian college students (*M*_age_ = 22.56 ± 2.87; 63.5% females). Among the main findings, the strongest (and positive) predictor of Studyholism is regression (maladaptive defense), while for Study Engagement, it is task-orientation (adaptive defense). Hence, Studyholism might be defined as a new potential clinical condition. Additionally, a critical analysis of all the defense mechanisms predicting Studyholism supports the appropriateness of the OCD-related framework for conceptualizing Studyholism. Regarding Study Engagement, even if generally associated with a positive defense style, the finding that it is positively predicted by projection confirms previous studies suggesting that, for some students, it might constitute a coping strategy with paranoid symptoms (and social anxiety and anxiety). Hence, we recommend screening engaged students for social impairment and clinically relevant symptoms that might be hidden by hard studying.

## 1. Introduction

Loscalzo and Giannini [1], referring to their workaholism model [2], proposed a comprehensive model of Studyholism (or obsession towards study) as a different construct than Study Addiction [3], even if both are related to problematic overstudying. In fact, among the main points of criticism that Loscalzo and Giannini [1] raised against Atroszko et al.’s [3] conceptualization, there is the assumption of an analogy with the construct of problematic overworking [3] that supported their adaptation of the Bergen Work Addiction Scale (BWAS) [4] to study behaviors by replacing the words “work” and “working” with “study” and “studying”. Loscalzo and Giannini [1] believe instead that, despite some similarities, there might be critical differences between work and study behaviors, requiring two different theorizations (and instruments) for problematic overworking and overstudying, as confirmed by recent studies [5,6,7].

Another critical difference between Study Addiction [3] and Studyholism [1] concerns their framework. Atroszko et al. [3] defined Study Addiction as a behavioral addiction characterized by the seven core components of substance addictions. Loscalzo and Giannini [1] referred instead to the Heavy Study Investment (HSI) framework since they aimed not to overpathologize a common behavior such as studying, in line with Billieux et al.’s [8] argumentations. HSI, namely heavy investment of time and energy in studying, is a concept introduced by Loscalzo and Giannini [1] referring to Snir and Harpaz’s [9] Heavy Work Investment model. More specifically, Loscalzo and Giannini [1] theorized that HSI might take three different forms: Disengaged Studyholism (i.e., students have high levels of Studyholism and low levels of Study Engagement), Engaged Studyholism (i.e., students have high Studyholism but also high Study Engagement), and Study Engagement (i.e., students have low Studyholism and high Study Engagement). Therefore, two HSI types are related to Studyholism (or obsession toward study), while one HSI type is related to Study Engagement and, therefore, it should not be labeled as a problematic studying behavior. Concerning Study Engagement, Loscalzo and Giannini [1] referred to the definition by Schaufeli et al. [10], which arose from that of work engagement, based on the assumption that students’ activities could be considered as work [11]. Therefore, Study Engagement has been conceptualized as a study behavior characterized by vigor, dedication, and absorption [10]. Besides these three dimensions, Loscalzo and Giannini [1] included intrinsic motivation as an additional component for the analysis of Study Engagement when analyzing Studyholism.

The studies conducted until now have supported the need for further analyzing Studyholism in youths since it is associated with adverse outcomes in many functional areas: academic, physical, psychological, and social well-being (e.g., [7,12]). In addition, the current literature suggests that Studyholism might be defined as a clinical condition, and more specifically as an obsessive compulsive (OCD)-related disorder (or, more generally, as an internalizing disorder) (e.g., [7,12]) rather than an addiction [3] (or externalizing disorder). Moreover, it is crucial to analyze Study Engagement in youths—even if generally associated with positive outcomes, such as higher grade point average and positive affect and lower dropout intention [12,13,14]—because it is related to social impairment due to study [12]. In addition, Loscalzo and Giannini recently showed that it is positively predicted by social anxiety [15], as well as by anxiety and paranoid ideation [7], suggesting that it could represent a coping strategy with these types of distressing symptom (similarly to work engagement for somatic symptoms [6]). Thus, some immature defenses may be present even in Study Engagement. Therefore, the present study aims at analyzing the defense mechanisms of Studyholism and Study Engagement to shed light on the defense profile that characterizes these two types of HSI. This research will provide further insight into the conceptualization of Studyholism as a clinical disorder (if associated with maladaptive defenses) and some information concerning the applicability of the OCD-related model to Studyholism, based on the available knowledge concerning OCD typical features [16].

The results about the defenses’ profile of HSI are important since clinical interventions might be tailored based on the defenses that characterize the person [17]. For instance, Bond [18] suggested that the prevalence of reaction formation and altruism might indicate to prompt the person to work as a volunteer, while people predominantly using acting-out might benefit from a consistent therapeutic intervention. Moreover, defense mechanisms might be changed through interventions. Albucher et al. [19] showed that after receiving 7-week group behavior therapy, OCD patients reported higher use of adaptative defenses, even if they did not show a lower use of immature defense styles (maladaptive, image-distorting, neurotic self-sacrificing styles). However, the analysis of the single defense mechanism of undoing (as an immature defense style) showed a statistically significant reduction. Interestingly, the intervention has been effective in improving the defense style even if not specifically addressing defense mechanisms and even if not belonging to the psychoanalytic approach, where the concept of defense mechanisms was first introduced.

### 1.1. Defense Mechanisms across Different Clinical Diagnoses

The concept of defense mechanisms is one of the core contributions of psychoanalysis [20,21]. Freud is the first one who talked about this unconscious process: he pointed out that psychopathology is associated with repression, which is an Ego defense mechanism. Since then, many other defenses have been described (e.g., denial or rationalization). However, besides differences in the specific mechanism of functioning, all of them aim to protect the person from excessive anxiety, hence protecting the self and the self-esteem of the subject. Everybody in everyday situations uses them since they are activated by threatening or anxiety-provoking circumstances [22], and they serve to manage stress and negative feelings [23]. However, defense mechanisms become pathological when the person uses them excessively [23] or uses immature defenses [24]. In fact, in line with the increasing number of defense mechanisms proposed, some authors—like Perry and Vaillant—suggested grouping them in higher-level categories (or defense styles), such as based on their maturity or immaturity levels [23]. In sum, defense mechanisms are part of everyday functioning, and using mature defenses is associated with positive outcomes, including higher self-esteem and self-confidence. On the other hand, the excessive use of defenses or the use of immature defenses is associated with adverse outcomes, including psychopathology. However, while the research evidence is consistent in showing that psychopathology is associated with higher use of immature defenses and less use of mature defenses, the results are inconsistent about the presence of an association between a specific defense (or a few specific defenses) and a particular clinical diagnosis, since people with a clinical diagnosis use several different defenses [23].

#### 1.1.1. Obsessive-Compulsive Disorder

The findings are inconsistent about the defense style characterizing the Obsessive-Compulsive Disorder (OCD). Kennedy et al. [25] used the 88-item version of the Defense Style Questionnaire (DSQ) [26], which allows evaluating 25 defense mechanisms and four defense styles: (i) immature/maladaptive (e.g., withdrawal, regression); (ii) image-distorting (e.g., omnipotence, splitting); (iii) neurotic self-sacrificing (e.g., reaction formation, pseudo-altruism); and (iv) mature/adaptive (e.g., suppression, sublimation, humor). Focusing on the four defense styles, rather than on the single defenses, Kennedy et al. [25] showed that people with OCD showed only a trend for higher use of maladaptive defenses when compared to a healthy control group, and no statistically significant difference concerning image-distorting, neurotic self-sacrificing scale, and adaptive styles.

Other studies concerning the defense style of OCD used the 40-item version of the DSQ [27]. This allows evaluating 20 defense mechanisms and three defense styles: (i) mature (e.g., sublimation, anticipation); (ii) neurotic (e.g., undoing, reaction formation); and (iii) immature (e.g., projection, acting-out). Atmaca et al. [28] showed that OCD patients, when compared to a healthy group, have lower scores on the mature defense style, while they have higher scores both on the neurotic and immature defense styles. Similarly, Shabanpour et al. [29] found that patients with OCD use more immature and less mature defenses than the healthy control group, suggesting that immature defenses might play a role in the development of the disorder. However, in contrast with Atmaca et al. [28], the OCD group does not differ from the control group on the neurotic style, even if there is a difference concerning a single neurotic defense, that is, higher idealization. Moreover, OCD patients have lower sublimation and humor (mature defenses) and higher projection, acting-out, devaluation, autistic fantasy, splitting, and rationalization (immature defenses) than the control group. Shabanpour et al. [29] highlighted that their results align with and extend previous findings showing higher use of acting-out and projection in OCD patients [30]. Finally, Blaya et al. [17] showed that OCD patients use to a greater extent immature defenses (in line with both Atmaca et al. [28] and Shambanpour et al. [29]) and neurotic defenses (in line with Atmaca et al. [28], but in contrast with Shambanpour et al. [29]) compared to a control group. However, they do not use mature defenses at a lower level (in line with Kennedy et al. [25] and in contrast with both Atmaca et al. [28] and Shambanpour et al. [29]). Regarding the single defense mechanisms, OCD patients have higher levels of pseudo-altruism (neurotic factor), projection, passive aggression, acting-out, and autistic fantasy (immature factor) than the control group [17]. Hence, the only defenses in which both Blaya et al. [17] and Shambanpour et al. [29] found a difference is for projection and acting-out (as also found by Pollock and Andrews [30]). Regarding acting-out, Blaya et al.’s [17] discriminant analysis found that it is the defense characterizing OCD and distinguishing it from the diagnosis of depression, social phobia, and panic disorder. The authors explain that acting-out might be an outcome of OCD symptoms: the person, when experiencing anxiety due to an obsessive thought, put in place a compulsive behavior, which corresponds to acting-out.

There are two other studies deserving a mention [20,31]. Offer et al. [20] used a 97-item self-report scale [32] that evaluates a lower number (i.e., eight) of defenses than the DSQ and a semi-structured interview assessing 11 defenses based on the interviewer’s evaluation [33]. Using the self-report scale, they found that OCD patients differ from the control group only on regression; however, they differ instead on all the defenses evaluated by the semi-structured interview (i.e., regression, denial, projection, introjection, reaction formation, undoing, displacement, intellectualization, compensation, sublimation, and repression). More specifically, the OCD group scores higher on all the defenses except for sublimation, where they score lower than the control group. Additionally, Offer et al. [20] suggest that major psychiatric disorders might share many defenses in common, such as regression, projection, denial, and repression. Hence, this study provides support for the role of regression in OCD; however, since the DSQ-40 [27] does not assess this defense mechanism, it is not possible to compare it with previous studies based on this scale [17,28,29], nor with the study by Kennedy et al. [25], because they did not analyze single defense mechanisms. Finally, Rubino et al. [31] performed a study involving a tachistoscope procedure [34] that allows coding reaction formation, barrier isolation, whitening isolation, disappearance of the threat, and absence of the threat. Their results showed that reaction formation and barrier isolation are used more often by OCD patients than by the control group. Hence, compared to previous studies, they added evidence concerning reaction formation and isolation, which have not been detected as defense mechanisms that differ between OCD patients and healthy controls by studies using the DSQ [17,25,28,29].

#### 1.1.2. Substance and Internet Addictions

Regarding the defense mechanisms in Substance Use Disorders (SUD), the results are again inconsistent across studies, also due to different types of SUD (i.e., alcohol, stimulants, opioids). Moreover, there are some similarities between OCD and SUD defense mechanisms (e.g., projection and acting-out). Among the studies using the DSQ-40 [27], Evren et al. [35] focused on male alcohol dependents and showed that the SUD group differed from the control group only on the neurotic defense style (higher score for the SUD group). However, there are differences in single defense mechanisms for the other two defense styles: higher projection, acting-out, splitting, and somatization (immature defenses) and lower humor (adaptive defenses). Moreover, their two logistic regression models showed that an immature defense style is a predictor of SUD, as well as acting-out, splitting, and (negatively) humor. In another study by Evren et al. [36], focusing this time on male heroin dependents, it has been found that, when compared to a healthy control group, heroin dependents score higher on the immature factor; they also score higher on the following defense mechanisms: idealization (neurotic defense), projection, acting-out, autistic fantasy, dissociation, and splitting (immature defenses). Then, in the two logistic regression models, immature defense style, devaluation, and splitting are predictors of SUD. While devaluation is a negative predictor, the others are positive predictors. Hence, Evren et al.’s studies [35,36] showed that, based on the specific substance of addiction, there might be differences in the defense style and mechanisms used. Nevertheless, projection, acting-out, and splitting seem to characterize both alcohol- and heroin-addicted people—compared to a control group—and the immature defense style predicts both types of addiction. It is interesting to compare these results with those from Raketic et al. [37], as they used two separate samples of women addicted to alcohol and opiates, and they showed that the two SUD groups did not differ from the healthy group on mature/adaptive defenses. However, while the alcohol-addicted women prevalently use neurotic defenses (such as in Evren et al. [35]), opiate addicts prevalently use immature defenses (such as in Evren et al. [36]). In addition, regarding single defense mechanisms, alcohol-addicted women mostly use pseudo-altruism, idealization, and undoing (neurotic defenses), while opiate-addicted women primarily use autistic fantasy, isolation, devaluation, denial, and splitting (immature defenses). Hence, the single defense mechanisms used by the alcohol-dependent group differ from Evren et al. [35], while there are just a few defenses (i.e., autistic fantasy and splitting) that have been found in the opiate group both in Raketic et al. [37] and Evren et al. [36]. Regarding cannabis addiction, the results found by Grebot and Dardar [38] are much different from the previous ones concerning alcohol and opioid addiction. Grebot and Dardar [38] showed that cannabis addiction is characterized by higher use of sublimation (mature defense) and lower use of displacement (immature defense).

Finally, a recent study [39] used the Response Evaluation Measure (REM-71) [40], which is a self-report evaluating 21 single defense mechanisms and two styles: maladaptive and adaptive defenses. They involved three different types of SUD: alcohol, cocaine (stimulant), and heroin (opioid). When comparing the SUD group (all types merged) with the control group, it showed higher levels of acting-out, fantasy, omnipotence, projection, and undoing (maladaptive defenses), higher levels of sublimation, and lower levels of intellectualization (adaptive defenses). Concerning differences between the three SUD groups, Taurino et al. [39] concluded that stimulants and opioid addictions are both characterized by a defense pattern comprehending the use of acting-out, fantasy, and sublimation. Instead, the subjects addicted to alcohol have a more maladaptive style, as they use a broader set of defenses: acting-out and fantasy, but also omnipotence, projection, and undoing. Interestingly, Taurino et al. [39], even recognizing that Grebot and Dardar [38] previously found higher levels of sublimation in SUD, suggest that this finding could be due to a methodological issue concerning the REM-71 sublimation scale, which has low internal reliability. However, besides the methodological issues of the two studies (non-clinical sample for Grebot and Dardar [38] and low reliability of the REM-71 scale for Taurino et al. [39]), two studies, using two different scales, find some evidence for higher sublimation—an adaptive defense—in SUD.

Finally, considering the literature about Internet Addiction Disorder (IAD), the situation is not more precise, as even the adaptive style contributes positively to IAD, and there are critical differences in the predictors compared to substance addiction findings. However, it should be noted that IAD is not yet recognized formally as a clinical disorder nor as a behavioral addiction, in contrast with Gambling Disorder, which has been formally recognized as a behavioral addiction [16]; hence, this does not allow us to consider the results about IAD as part of the SUD and behavioral addiction literature.

The two studies using the DSQ-88 and performing a path analysis [41,42] highlighted that the defense style is a good predictor of IAD, with all the four DSQ-88 styles contributing positively (even if the maladaptive style and the image-distorting style have the highest contribution, and the path for the adaptive style, in one of the two studies [42], is lower than 0.20). In a subsequent study, Floros et al. [43] provided an insight into the role of the single defense mechanisms in IAD by highlighting through a discriminant analysis that IAD students might be distinguished from non-IAD students based on their higher help-rejecting complaints and lower sublimation. Moreover, IAD students differ from a healthy control group on maladaptive style (higher in IAD), sublimation (lower in IAD), and help-rejecting complaining (higher in IAD). Finally, a study conducted using the DSQ-40 by Waqas et al. [44] found (generally low) negative correlations between IAD and sublimation and rationalization, and positive correlations with projection, denial, devaluation, somatization, autistic fantasy, splitting, passive aggression, and displacement. Moreover, a multiple linear regression showed that sublimation is a negative predictor of IAD, while denial, autistic fantasy, passive aggression, and displacement are positive predictors. However, the beta values are again low, ranging between 0.09 (sublimation and denial) and 0.17 (autistic fantasy).

### 1.2. The Present Study

This study aims to analyze the defense profile of Studyholism and Study Engagement, which are two types of HSI, with the greatest detail possible. Therefore, we used the DSQ-88 to analyze 25 single defense mechanisms. More specifically, we have these objectives: (i) to explore which defense mechanisms predict Studyholism and Study Engagement; (ii) to analyze if there are differences in the defense mechanisms between students characterized by high/low levels of Studyholism/Study Engagement.

This is the first study concerning defense mechanisms in problematic overstudying and Study Engagement; moreover, there is no clear link between specific clinical diagnoses and defenses profiles. *Therefore, we cannot posit specific hypotheses*. However, based on previous research (e.g., [12]) we have a general expectation concerning a more dysfunctional/immature defense style in Studyholism (e.g., regression and somatization as positive predictors) and a more adaptive style in Study Engagement (e.g., task-orientation as a positive predictor and projective identification as a negative predictor), even if we do not exclude the possibility of some immature defenses also characterizing Study Engagement.

Since the literature concerning OCD, SUD, and IAD is inconsistent, it is not possible to use our results for shedding light on the internalizing and/or externalizing nature of Studyholism. However, it will allow us to obtain some information concerning the applicability of the OCD-related model to Studyholism, based on the available knowledge concerning OCD typical features [16].

Regarding the analyses we conducted to address our objectives, we first performed a SEM model (more specifically, a path analysis) with the single defense mechanisms as predictors of Studyholism and Study Engagement. Next, concerning our second aim, we performed four MANOVAs to evaluate differences in the 25 single defense mechanisms (plus the lie/control scale) and the three defense styles between students characterized by high/low levels of Studyholism/Study Engagement. Finally, to analyze further the role of defense mechanisms as predictors of Studyholism and Study Engagement (first aim), we performed a non-parametric analysis. More specifically, we conducted four binary logistic regressions using as predictors the 25 single defense mechanisms and high and low Studyholism/Study Engagement as the dichotomous outcome.

## 2. Materials and Methods

### 2.1. Participants

We gathered 422 Italian college students aged between 18 and 47 years (*M*_age_ = 22.56 ± 2.87; 63.5% females). Most of the students were either engaged (52.8%) or single (45.0%). There were just a few cohabiting (1.9%) or married (0.3%) participants. Concerning their professional status, most participants did not work besides studying (87.7%). The students attended their courses in north or central Italy (i.e., Florence, Bologna, Ferrara, or Venice). The areas of study most represented are Psychology (23.9%) and Engineering (16.8%). However, there are students from other majors: Design (8.3%), Health Professions (7.3%), Law (6.9%), Architecture (6.6%), Medical studies (5.9%), Literature and Philosophy (5.5%), Economy (5.5%), Chemical studies (4.7%), Math and Physics (4.6%), and Social and Political Sciences (4.0%). Finally, concerning the year of study, the percentages from first to fifth year are 14.9%, 5.5%, 23.2%, 23.9%, and 32.5%, respectively.

Regarding study-related variables, most participants declared usually studying on the weekend (86.3%), and a minority said to have repeated at least a school year (21.8%). Concerning the time spent studying generally, the hours per day ranged between 0 and 14 (*M* = 4.31 ± 2.12), and the days per week ranged between 0 and 7 (*M* = 5.16 ± 1.27). When considering the time spent studying before exams, the *Mean* value was 7.44 ± 2.48 (range 2–18) for hours per day, and 6.47 ± 0.80 (range 1–7) for days per week. Finally, the grade point average ranged between 21 and 30, with a *Mean* of 27.06 ± 2.09.

### 2.2. Materials

#### 2.2.1. Studyholism Inventory (SI-10)

The SI-10 [14] is a 10-item self-report instrument made up of two scales: Studyholism and Study Engagement. Each scale comprises four items (plus a filler item). The SI-10 also has a head-sheet with questions about study habits (e.g., studying on the weekend, time spent studying). The participants fill the scale through a 5-point Likert scale ranging between 1 (*Strongly Disagree*) and 5 (*Strongly Agree*). The SI-10 is currently available in Italian, Polish, Croatian, Spanish, Indonesian, and English. For the present study, we administered the Italian version. The alpha values are good for both the SI-10 scales: Studyholism, 0.84; Study Engagement, 0.81 [14].

#### 2.2.2. Defense Style Questionnaire (DSQ)

The DSQ [26] is an 88-item self-report scale for the assessment of defensive mechanisms. This scale was developed by Bond et al. [26]. It assesses, using between one and nine items, 25 defense mechanisms: acting-out, affiliation, undoing, anticipation, passive aggressive, consumption, denial, fantasy, reaction formation, primitive idealization, projective identification, inhibition, isolation of affect, help-rejecting complaining, omnipotence, task-orientation, projection, pseudo-altruism, regression, suppression, withdrawal, splitting, somatization, sublimation, and humor. Moreover, there is also a Lie (or control) scale, which allows evaluating the respondent’s tendency to provide a false profile of him/herself. The response format of the DSQ is a 9-point Likert scale ranging between 1 (*Totally Disagree*) and 9 (*Totally Agree*). We administered the Italian version of the DSQ [21]. For scoring purposes, after a careful review and discussion among the authors, we moved item 57 (“I would be very nervous if an airplane in which I was flying lost an engine”) from the denial scale (as suggested in Appendix 2 by San Martini et al. [21]) to the lie scale. The content of the item clearly belongs to the lie scale (where the Appendix reports including the item “5 7” instead of “57”). Moreover, following San Martini et al.’s [21] analyses, we created the following macro-groups, or three defense styles, which are different from the original version [26]: (i) Maladaptive style, comprehending regression, acting-out, projection, somatization, passive-aggression, withdrawal, fantasy, consumption, help-rejecting complaining, projective identification, and undoing (for a total of 37 items); (ii) Image-Distorting style, made up of denial, omnipotence, isolation of affect, and splitting (for a total of 16 items, and not 17, as written in the original paper [21]; (iii) Adaptive style, comprising anticipation, task-orientation, pseudo-altruism, primitive idealization, sublimation, suppression, and humor (for a total of 13 items, instead of 12, as written, probably by mistake, in the original paper [21]). However, the authors themselves state that the Image-Distorting and the Adaptive style should be improved, as their psychometric properties are not as good as for the Maladaptive style scale [21]. Hence, considering this methodological issue, in the current study, we use the three defense styles only for multivariate analyses of variance (and not for the path analysis and logistic regression models). Additionally, since the Italian scales are different from the original ones, we cannot compare our results with other studies using the DSQ-88 defense styles. Concerning the internal reliability of the single defense mechanisms, the lowest alpha values are 0.16 (consumption), 0.26 (suppression) and 0.27 (denial), while the highest values are 0.71 (omnipotence) and 0.69 (acting-out). There are four single-item scales for which it is not possible to calculate internal reliability. The alpha values for all the other scales range between 0.32 and 0.67 [21]. The internal consistency values for the three defense styles are: Maladaptive style, 0.85; Image-Distorting style, 0.72; Adaptive style, 0.57 [21].

### 2.3. Procedure

First, we obtained study approval from the Ethical Committee of the University of Florence. Next, students were contacted at their universities, in common spaces such as libraries and university rooms outside classes. Each participant signed the informed consent form before filling out the paper-and-pencil questionnaire, which included a first page asking for demographic variables (e.g., gender, age), the SI-10, and the DSQ. All the data were gathered before the COVID-19 outbreak.

### 2.4. Data Analysis

We performed the analyses through SPSS.27 (Chicago, IL, USA) and AMOS.20 (Chicago, IL, USA).

First, we analyzed the variables’ descriptive statistics (including skewness and kurtosis). Next, we analyzed the zero-order correlations between Studyholism, Study Engagement, and defense mechanisms (including the three defense styles). Then, we performed a SEM (more specifically, a path analysis using the Maximum Likelihood estimate method) with the single defense mechanisms as predictors of Studyholism and Study Engagement. The cut-off values provided by Byrne [45], Hu and Bentler [46], and Reeve et al. [47] have been used as a reference for the evaluation of the fit of the model. For these analyses, *p* < 0.05 is considered statistically significant.

Next, we performed four MANOVAs to evaluate differences in the DSQ scales (i.e., 25 single defense mechanisms and lie scale) and the three DSQ defense styles between students characterized by high/low levels of Studyholism/Study Engagement. The high/low levels of Studyholism/Study Engagement groups have been created referring to the SI-10 cut-off values for Italian college students [14]. Since our MANOVAs foresee a total of 58 follow-up analyses, we adjusted the alpha level through the Bonferroni correction for multiple comparisons. Hence, we set an adjusted alpha level of 0.001 [48].

Finally, to further analyze the role of defense mechanisms as predictors of Studyholism and Study Engagement, we performed eight binary logistic regressions using as predictors: (i) the 11 single defense mechanisms included in the maladaptive style; (ii) the 4 single defense mechanisms included in the image-distorting style; (iii) the 7 single defense mechanisms included in the adaptive style; and (iv) the 3 defenses that do not belong to any of the three defense styles. We performed these four models separately for Studyholism and Study Engagement. The dichotomous outcome of these binary logistic regressions is high and low Studyholism/Study Engagement, respectively.

## 3. Results

### 3.1. Preliminary Analyses

As a first step, we analyzed the descriptive statistics of all the study variables (see Table 1). The number of items varies between the different defense mechanisms (from single-item scales to nine items); therefore, the range changes among the 25 defense mechanisms. The normality assumption is fulfilled for all the scales (including Studyholism, Study Engagement, the DSQ Lie scale, and the three defense styles), except for projective identifications and projections, which showed positive (and higher than 1) values for both skewness and kurtosis. However, referring to the content of the scales, we might expect a distribution characterized by a higher proportion of low scores (as indicated by positive skewness) and by most of the participants’ scores around the *Mean* (as indicated by positive kurtosis) in a non-clinical sample of college students. Regarding the inclusion of these two scales in the path analysis, Bentler [49] suggests that values higher than five indicate that data are not normally distributed, and the values for these two defense mechanisms are lower than this cut-off. Therefore, we used them in the subsequent analyses.

Then, we calculated the zero-order correlations between Studyholism and Study Engagement and the DSQ scales, including the three defense styles (see Table 2). Regarding Studyholism, we found statistically significant correlations with all the defense mechanisms, except for affiliation, denial, reaction formation, isolation of affect, sublimation, and humor (for a total of 19 statistically significant correlations). Besides the negative correlation with omnipotence and suppression, all the correlations are positive. Additionally, the values of correlation are generally low. The highest values are for regression (0.52), somatization (0.41), and withdrawal (0.40), which are maladaptive defense styles. Regarding Study Engagement, the number of statistically significant correlations is lower (11 up to the 25 defenses). There is a positive correlation with task-orientation (which corresponds to the highest *r* value: 0.35) and anticipation, which are adaptive defense mechanisms. All the other values of correlations are negative. Concerning the Lie scale, it correlates positively with Studyholism and negatively with Study Engagement.

### 3.2. Defense Mechanisms as Predictors of Studyholism and Study Engagement

We ran a path analysis model with the 25 defense mechanisms as Studyholism and Study Engagement predictors. The model showed an excellent fit to the data: CFI = 0.999; GFI = 0.999; RMSEA = 0.054 (C.I. 90% = 0.000–0.132); *χ*^2^ = 4.443, df = 2, *χ*^2^/df = 1.72, *p* = 0.108. Moreover, the defense mechanisms explain a good percentage of the variance for both Studyholism (42.7%) and, to a lower extent, Study Engagement (31.3%). Studyholism is predicted by a higher number of defenses (i.e., 12) than Study Engagement (i.e., 6); however, some Studyholism predictors have low *β* values, and for three variables the value is lower than 0.10. The strongest (positive) predictor of Studyholism is regression (a maladaptive defense), while the strongest (positive) predictor of Study Engagement is task-orientation (an adaptive defense). Table 3 shows the standardized path weight (and *p* values) for all the statistically significant predictors.

### 3.3. Differences in Defense Mechanisms between Students with High and Low Levels of Studyholism and Study Engagement

To conduct MANOVAs with high and low levels of Studyholism and Study Engagement as the independent variables, we created—using the cut-off values for high and low Studyholism/Study Engagement [9]—the following four groups of student: high Studyholism (*n* = 24, 5.7%), low Studyholism (*n* = 89, 21.1%), high Study Engagement (*n* = 53, 12.6%), and low Study Engagement (*n* = 39, 9.2%).

Then, we ran the MANOVAs with the defense mechanisms and the Lie scale as dependent variables. Regarding Studyholism, the multivariate test highlighted a statistically significant effect on the DSQ variables: *F*(26,81) = 8.94, *p* < 0.001, η^2^ = 0.74. More specifically, follow-up ANOVAs (using the adjusted alpha level of 0.001) showed statistically significant differences in the following defense mechanisms: acting-out, projective identification, help-rejecting complaining, projection, regression, withdrawal, splitting, and somatization. Follow-up ANOVA is also statistically significant for the Lie scale: students with high Studyholism try giving a better image of themselves than students with low Studyholism. Despite this, they also score higher than their peers on the aforementioned defense mechanisms.

Regarding Study Engagement, the multivariate test highlighted a statistically significant effect: *F*(26,64) = 4.63, *p* < 0.001, η^2^ = 0.65. More specifically, follow-up ANOVAs (using the adjusted alpha level of 0.001) showed statistically significant differences in the following defense mechanisms: acting-out, passive aggression, projective identification, isolation, and task-orientation. Students with high Study Engagement score lower on all the defense mechanisms than their peers with low Study Engagement, except for task-orientation, where the score is higher for the high Study Engagement group.

**Figure 1:**
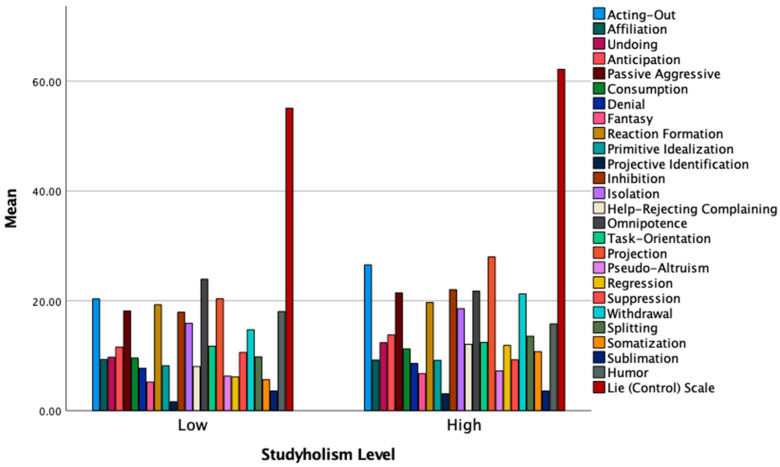
Mean levels of the defense mechanisms and the Lie scale by low and high Studyholism.

**Figure 2:**
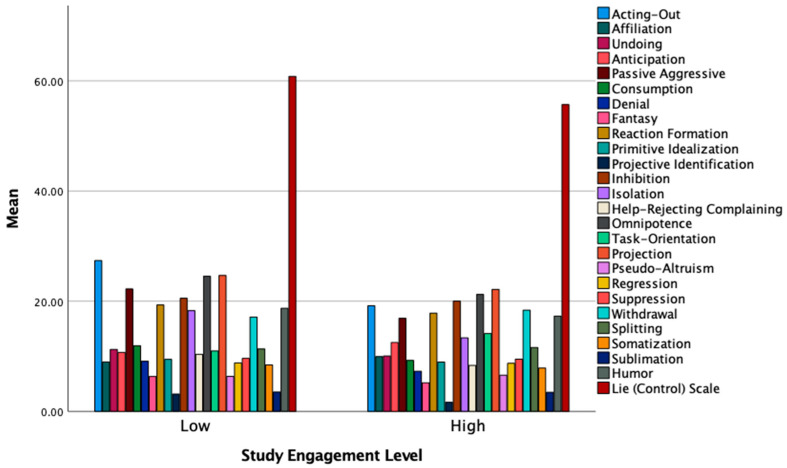
Mean levels of the defense mechanisms and the Lie scale by low and high Study Engagement.

Table 4, Table 5, Table 6 and Table 7 shows the results of follow-up ANOVA analyses grouped referring to San Martini et al.’s [21] macro-groups: Maladaptive style, Image-Distorting style, Adaptive style, and a last group including the lie/control scale and the three defenses not belonging to any of the previous factors. Moreover, Figure 1 and Figure 2 graphically show the *Mean* differences on the single defense mechanisms between low and high Studyholism/Study Engagement.

**Figure 3:**
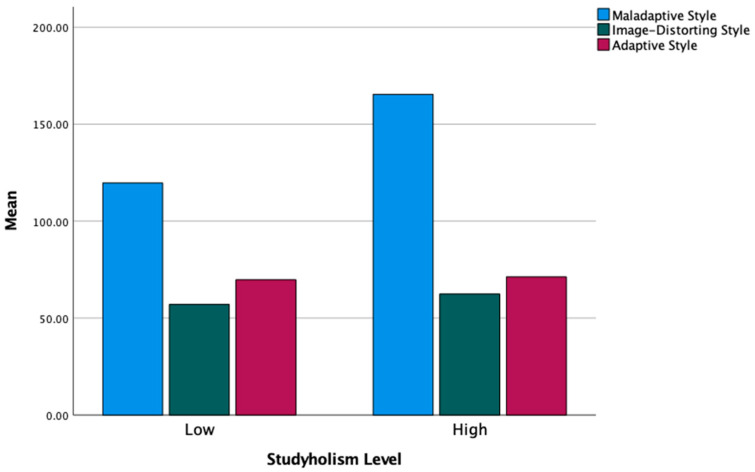
Mean levels of the three defense styles by low and high Studyholism.

**Figure 4:**
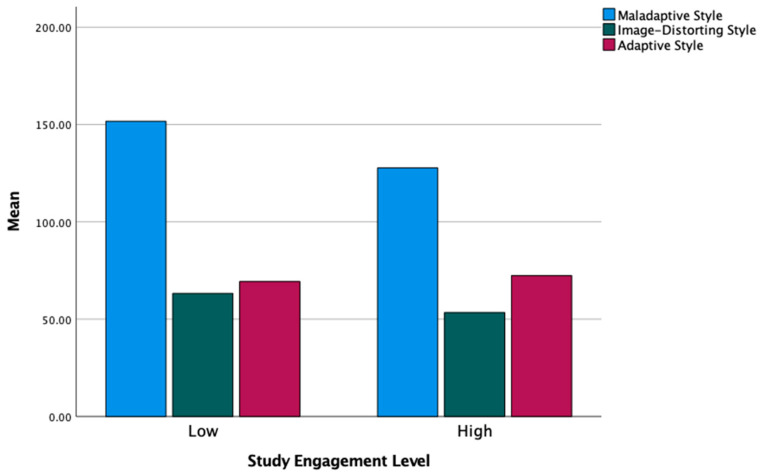
Mean levels of the three defense styles by low and high Study Engagement.

Finally, we ran the MANOVAs with the three defense styles as the dependent variables. The multivariate test is statistically significant for both Studyholism (*F*(3,105) = 22.32, *p* < 0.001, η^2^ = 0.39) and Study Engagement (*F*(3,87) = 7.09, *p* < 0.001, η^2^ = 0.20). However, the follow-up ANOVAs highlighted statistically significant differences only for the Maladaptive style: the higher scores are for the high Studyholism group and the low Study Engagement group. Table 8 shows the results of follow-up ANOVAs analyses. Figure 3 and Figure 4 graphically show the *Mean* differences in the three defense styles between low and high Studyholism/Study Engagement.

### 3.4. Defense Mechanisms as Predictors of High Studyholism

We ran four binary logistic regressions with the single defense mechanisms included in the models accordingly to their inclusion in the maladaptive, image-distorting, adaptive, or any factor.

The model with the 11 maladaptive defense mechanisms is statistically significant (LR Test = *χ*^2^(11) = 92.77, *p* < 0.001) and explains a large variance in the probability of being “diagnosed” as having high Studyholism: Nagelkerke’s R = 0.874. In fact, using the 11 defense mechanisms, the percentage of students properly classified as having high and low Studyholism changes from 78.4% (baseline model) to 95.5%. Regarding the statistically significant (positive) predictors, they are regression (OR = 2.68 (CI95% 1.31–5.46); *p* = 0.007), somatization (OR = 2.81 (CI95% 1.26–6.27); *p* = 0.012), and (marginally) projective identification (OR = 3.71 (CI95% 0.99–13.93); *p* = 0.052). The model with the four image-distorting defense mechanisms is statistically significant (LR Test = *χ*^2^(4) = 18.10, *p* = 0.001); however, it explains a low variance in Studyholism: Nagelkerke’s R = 0.232. In fact, the percentage of students properly classified changes to 82.0%, and there is a statistically significant (positive) predictor only, namely splitting: OR = 1.24 (CI95% 1.09–1.41); *p* < 0.001.

Regarding the seven adaptive defense mechanisms, the model is again statistically significant but with a low percentage of variance explained: LR Test = *χ*^2^(7) = 25.26, *p* < 0.001; Nagelkerke’s R = 0.223. In line with this, the percentage of students correctly classified is 78.6%. The statistically significant positive predictors are anticipation (OR = 1.29 (CI95% 1.04–1.59); *p* = 0.019) and pseudo-altruism (OR = 1.64 (CI95% 1.02–2.62); *p* = 0.040). Moreover, humor is a statistically significant negative predictor (OR = 0.98 (CI95% 0.78–0.99); *p* = 0.035). Finally, the model with the three defense mechanisms not included in a defense style, namely affiliation, inhibition, and reaction formation, is not statistically significant: LR Test = *χ*^2^(3) = 5.05, *p* = 0.168; Nagelkerke’s R = 0.069, even if inhibition showed statistical significance: OR = 1.07 (CI95% 1.01–1.14); *p* = 0.029.

### 3.5. Defense Mechanisms as Predictors of High Study Engagement

We repeated the previous four binary logistic regressions, using high and low Study Engagement as the dichotomous outcome variable.

The model with the 11 maladaptive defense mechanisms is statistically significant (LR Test = *χ*^2^(11) = 52.08, *p* < 0.001) and explains a good variance in the outcome variable: Nagelkerke’s R = 0.586. The percentage of students properly classified as having high and low Study Engagement changes from 57.6% (baseline model) to 75.0%. The statistically significant predictors are acting-out (OR = 0.83 (CI95% 0.73–0.95); *p* = 0.007), projection (OR = 1.13 (CI95% 1.01–1.26); *p* = 0.031), passive aggressive (OR = 0.88 (CI95% 0.78–0.99); *p* = 0.034), and projective identification (OR = 0.58 (CI95% 0.31–0.77); *p* = 0.002). Except for projection, the defense mechanisms are negative predictors. The model with the four image-distorting defense mechanisms is statistically significant: LR Test = *χ*^2^(4) = 19.31, *p* < 0.001. However, in line with the low value of Nagelkerke’s R (0.254), the percentage of students properly classified changes to 68.5% only. There are two statistically significant predictors: isolation, as a negative predictor (OR = 0.90 (CI95% 0.83–0.97); *p* = 0.005), and (marginally) splitting, as a positive predictor [OR = 1.12 (CI95% 1.00–1.23); *p* = 0.050].

Regarding the seven adaptive defense mechanisms, the model is again statistically significant but with a low percentage of variance explained: LR Test = *χ*^2^(7) = 26.45, *p* < 0.001; Nagelkerke’s R = 0.339. In line with this, the percentage of students correctly classified using these defense mechanisms is 69.2%. There is a statistically significant (positive) predictor, namely task-orientation: OR = 1.36 (CI95% 1.16–1.61); *p* < 0.001. Finally, the model with the three defense mechanisms not included in a defense style is not statistically significant: LR Test = *χ*^2^(3) = 3.46, *p* = 0.326; Nagelkerke’s R = 0.050.

## 4. Discussion

The current study aimed at analyzing the defense profile of Studyholism and Study Engagement (i.e., two types of Heavy Study Investment—HSI) with the greatest detail possible, hence analyzing 25 defense mechanisms. To the best of the authors’ knowledge, this is the first study about the defenses characterizing these two types of study behavior; therefore, we did not set specific hypotheses, even if we expected that Studyholism, as a new *potential* clinical diagnosis, would have been characterized by a more maladaptive defense style than Study Engagement. However, since previous studies showed that Study Engagement, even if generally associated with positive outcomes, is also a predictor of social impairment due to study (e.g., [12]) and might represent a coping strategy for social anxiety, anxiety, and paranoid ideation [7,15], we did not exclude the possibility of the presence of some maladaptive defenses even in Study Engagement.

First, correlation analyses showed that Studyholism correlates with most defense mechanisms. There is a positive correlation with all the maladaptive defense styles, with the highest value for regression and withdrawal. Additionally, it has a low positive correlation with splitting and a low negative correlation with omnipotence (image-distorting defenses). Regarding adaptive defenses, the correlation is statistically significant for almost all of them. However, besides the negative correlation with suppression, all the others are positive correlations (even if generally low). Finally, there is a positive correlation with inhibition, which does not belong to any cluster based on the Italian factor analyses [21]. Considering Study Engagement, it negatively correlates with most maladaptive defenses and a few image-distorting defenses. Additionally, it has a positive correlation with two adaptive defenses. The highest correlation value is for an adaptative defense, namely task-orientation. Finally, concerning the three main defense styles, Studyholism has a statistically positive (and high) correlation with the maladaptive style, while Study Engagement correlates, weakly and negatively, with the maladaptive and the image-distorting style, and positively with the adaptative style. Hence, these results provide preliminary evidence for a more maladaptive defense style in Studyholism and a more adaptive defense style in Study Engagement.

Next, the path analysis model indicated that defense mechanisms have an essential role in predicting HSI, especially Studyholism. They explain 42.7% of the variance in Studyholism—supporting its definition as a clinical condition [23,24]—and 31.3% of the variance in Study Engagement. In line with the expectation of a maladaptive defense style in Studyholism and an adaptive style in Study Engagement, the strongest predictors are, respectively, regression (*β* = 0.29) and task-orientation (*β* = 0.31).

Regarding the other statistically significant predictors of Studyholism, there are other maladaptive defense mechanisms (i.e., projective identification, help-rejecting complaining, withdrawal, and somatization) whose *β* values range between 0.11 and 0.17. Additionally, omnipotence is the only predictor of the image-distorting style, but it is negative. Concerning adaptive defenses, while suppression is a negative predictor, task-orientation and pseudo-altruism are positive predictors. Finally, even if the *β* values are very low (and in some cases only marginally statistically significant), anticipation is another positive predictor; moreover, affiliation (which might be defined as an adaptive defense, even if not belonging to any cluster accordingly to San Martini et al. [21]) and sublimation are negative predictors.

Regarding adaptive defenses, it is interesting to note that suppression concerns the ability to control thoughts; hence, it is a negative predictor in line with the obsessive nature of Studyholism, as suggested by the OCD-related conceptualization (e.g., [7,12,14,15]). Task-orientation is related to working hard to feel better; hence, its positive value in predicting Studyholism is again in line with the conceptualization of Studyholism as a clinical form of overstudying and as an OCD-related disorder, since overstudying represents the compulsion put in place to deal with distressing feelings or thoughts. Anticipation concerns the tendency to think about and plan an exam or job interview and the importance of predicting a negative situation to cope with it better. Again, its positive value aligns with the obsessive thinking that characterizes overstudying in Loscalzo and Giannini’s view. Affiliation concerns the tendency to seek help and affiliate with others; hence, this is a negative predictor in line with the social issues associated with Studyholism (e.g., [12,15]). Finally, pseudo-altruism is made up of one item only, which concerns the satisfaction in helping others and to be depressed if this would be avoided. We speculate that this might be a positive predictor since it resembles the tendency to give a better image of themselves, which appears to characterize Studyholism as being positively correlated with the control scale. In sum, these results seem to support the definition of Studyholism as an OCD-related disorder (or an internalizing disorder).

Moreover, omnipotence and sublimation are negative predictors of Studyholism, in contrast with the studies concerning SUD, which found that they have higher omnipotence and sublimation levels than a control group [38,39]. The results concerning lower sublimation and higher regression in Studyholics are in line with the studies concerning OCD [20,29] and, regarding lower sublimation only, with the studies about IAD [43,44]. Hence, we speculate that these results might suggest that Studyholism is characterized by a defense style that is more similar to OCD than to SUD, hence supporting its conceptualization as an OCD-related disorder. Additionally, as IAD is more similar to Studyholism than to SUD concerning sublimation, we could speculate that it might be useful to also take into account the presence of OCD features in this condition, in line with previous suggestions about avoiding a confirmatory approach based on addiction for defining excessive behaviors (e.g., [1,50]). In fact, it should be noted that IAD is not formally recognized as a behavioral addiction, in contrast with Gambling Disorder [16]. Though, it is important to bear in mind that the literature, until now, did not find a specific association between clinical diagnoses and single defense mechanisms; hence, it is not possible to use these data for definitive conclusions concerning the internalizing and/or externalizing nature of problematic overstudying.

Concerning Study Engagement, besides the strongest predictor (i.e., task-orientation), it is positively predicted by another adaptive defense (i.e., anticipation) and, negatively, by two maladaptive defense mechanisms (i.e., acting-out and projective identification) and reaction formation, which does not belong to any of San Martini et al.’s [21] defense styles, but that might be classified as a maladaptive/neurotic defense [27]. Hence, as expected, Study Engagement is predicted by a more mature defense style. However, it is interesting to note that it is also positively predicted by a maladaptive defense mechanism: projection. The content of the projection items deals, for example, with the feeling of being mistreated or that everybody is against the person, or with the person reporting that others tell him/her that he/she has a persecution complex. Hence, this result further supports Loscalzo and Giannini’s [7] finding that Study Engagement might constitute a coping strategy with paranoid (and anxiety) symptoms. Therefore, our study confirms that Study Engagement, even if generally associated with positive outcomes, should not be overlooked for the possible presence of negative outcomes (such as social impairment (e.g., [12]) and clinically relevant symptoms hidden by hard studying (such as social anxiety, anxiety, and paranoid ideation, [7,15]). Hence, from a methodological point of view, it also supports Loscalzo and Giannini’s [1] suggestions about distinguishing between Engaged and Disengaged Studyholics as two different forms of Studyholism. Moreover, it highlights the critical value of using an instrument that allows evaluating both Studyholism and Study Engagement (i.e., the Studyholism Inventory, SI-10 [14]), instead of a scale that allows evaluating Study Engagement (Utrecht Work Engagement Scale—student version) [51] or problematic overstudying (Bergen Study Addiction Scale—BStAS) [3] only. Additionally, based on their findings, Loscalzo and Giannini suggested that the BStAS might not properly distinguish between Study Engagement and study addiction (e.g., [14]), as another critical point concerning using the BStAS. Including both Studyholism and Study Engagement allows for controlling the effect of Study Engagement on Studyholism (and vice versa) in most analyses, such as path analysis, MANOVAs, and regression analysis. Therefore, it is important to measure both when analyzing HSI. In fact, Loscalzo and Giannini [1] suggest that Study Engagement might be present on its own (i.e., engaged student) but also be co-present in Studyholism (i.e., engaged Studyholic). Hence, also considering the negative aspects associated with Study Engagement, it is critical to include both in the analyses.

Next, we further analyzed the role of defense mechanisms in HSI by analyzing the *Mean* differences between students characterized by high levels of Studyholism/Study Engagement and low levels of the two forms of HSI. Hence, this might be considered a comparison between a “clinical” and “healthy” group concerning the variables under analysis. Moreover, through MANOVAs, we analyzed the impact of HSI on defense mechanisms; therefore, in contrast with the path analysis, HSI is the independent/predictor variable. Cramer [23] prompted scholars to analyze the relation between defenses and psychopathology since the available data do not establish if defenses lead to psychopathology or if psychopathology leads to the use of specific defenses. Additionally, it might be possible that the relation between these variables is circular or intrinsic.

Regarding Studyholism, MANOVA analyses showed that students with high Studyholism, compared to their peers with low Studyholism, have higher levels of splitting (image-distorting defense) and acting-out, projective identification, help-rejecting complaining, projection, regression, withdrawal, and somatization (maladaptive defenses). Hence, it is confirmed that Studyholism is associated with a more maladaptive defense style, suggesting it might be defined as a new *potential* clinical disorder [23,24]. As shown by the previous literature review, it is hard to use these findings to state if the defense asset of Studyholics is more similar to OCD or SUD, as the results are inconsistent. However, critically reflecting on each of the defenses, we can suggest that the OCD-related framework might be appropriate for Studyholism, even if we cannot exclude that a different explanation could be applied, as we do not have empirical data supporting our speculations. First, splitting refers to a “black and white” type of thinking (e.g., a person is totally good or totally bad); hence, this is in line with the rigidity and strict morality that usually characterizes OCD patients. Acting-out, as suggested by Blaya et al. [17], might be an outcome of OCD symptoms since the compulsive behavior in OCD represents the acting-out that arises from an experience of anxiety due to an obsessive thought. Additionally, this is in line with Loscalzo and Giannini’s [1] theorization about the possible presence of aggressive behaviors in Studyholics. Projective identification is a single-item scale concerning the assumption that someone is emotionally robbing a person of all she/he has. Hence, we speculate that this might be due to the maladaptive defense style characterizing OCD and, probably, the lack of insight that is sometimes present in OCD (the level of insight is an OCD specifier according to DSM-5 [16] criteria). Help-rejecting complaining concerns the belief that doctors cannot help the person eliminate the symptoms and that the person is not understood for her/his complaining. This seems to be in line with the obsessive nature of OCD, which leads the person to ruminate, and with the findings of the strong role of trait worry in predicting Studyholism (e.g., [12]). Besides being the strongest predictor of Studyholism, regression is also present at higher levels in students with a “diagnosis” of Studyholism compared to “healthy” peers. This evaluates the tendency to act childish when frustrated and fall apart under stress. Hence, it might represent the fact that OCD/Studyholic compulsion does not represent a realistic (or mature/adult) way of coping with distressing feelings. Withdrawal refers to the tendency to depart from people when offended and sad. This is in line with the negative predictive value of affiliation on Studyholism and the social issues associated with Studyholism (e.g., [12,15]) that could be linked to a lack of a proper social network to whom the person could refer in case of problems. Finally, somatization concerns the tendency to translate negative emotions into somatic symptoms, a feature of internalizing (including OCD) rather than externalizing disorders. In fact, externalizing disorders are characterized by expressing negative feelings towards the outside. Finally, it is interesting to note that subjects with high Studyholism tend to give a better image of themselves. Therefore, this might suggest a strong desire for social appreciation in this type of student, who uses overstudying, a type of behavior generally accepted by society (compared, for example, with the use of substances), to manage their emotional difficulties.

Regarding Study Engagement, we found that students with high levels of this variable score lower than their peers on acting-out, passive aggressive, projective identification (maladaptive defenses), and isolation (image-distorting defense), while they score higher on task-orientation (adaptive defense), in line with the general better defense functioning associated with Study Engagement.

In line with the findings concerning the single defense mechanisms, the MANOVAs conducted on the three defense styles (maladaptive, image-distorting, and adaptive styles) showed a statistically significant difference in the maladaptive style: the high Studyholism group and the low Study Engagement group score higher on this scale, hence confirming our general expectation of a more adaptive style in Study Engagement and a more maladaptive style in Studyholism.

Finally, we performed binary logistic regressions to analyze the predictive role of the single defense mechanisms in predicting a “diagnosis” of Studyholism and Study Engagement, that is, of being a student with high levels of Studyholism and Study Engagement, accordingly to their cut-off scores [14]. Regarding Studyholism, the model with the 11 maladaptive defenses explains a significant variance in the probability of being “diagnosed” as having high Studyholism (87.4%). More specifically, regression, somatization, and (marginally) projective identification are positive predictors. Additionally, splitting is another positive predictor concerning the model with the four image-distorting defense styles. Therefore, these results support the definition of Studyholism as a clinical condition since the excessive use of defense mechanisms, or the use of maladaptive defenses (as we found) is associated with psychopathology [23,24]. In addition, the model with the seven adaptive defenses showed that humor is a negative predictor, while anticipation and pseudo-altruism are positive predictors (in line with the path analysis results and with the conceptualization of Studyholism as an OCD-related disorder, as previously explained regarding these two defense mechanisms). The model with the three defenses not belonging to any defense style, according to San Martini et al. [21], is not statistically significant.

Regarding Study Engagement, maladaptive defenses are again good predictors of the outcome (58.6%), and the negative predictors are acting-out, passive aggression, and projective identification. Instead, in line with the path analysis model results, projection is a positive predictor. Regarding image-distorting defenses, isolation is a negative predictor, while splitting is a (marginally) positive predictor. Moreover, there is a positive predictor among the adaptive defenses, namely task-orientation. In sum, even if the generally better defense style of Study Engagement is confirmed, the results also support the need of screening engaged students for the presence of important clinical conditions, such as social anxiety, anxiety, and paranoid ideation, which might characterize some of them [7,15]. The model with the three defenses not belonging to any defense style is again not statistically significant.

Among the limitations of this study, there is a slightly higher prevalence of females (63.5%) among participants, a low representation of second-year students, and a lack of students from southern Italy. However, the sample is heterogeneous concerning the year and the major of study. Additionally, the instrument used to evaluate defense mechanisms [26] has a different factor structure in the Italian version [21] compared to the original one. More critically, the Image-Distorting and the Adaptive style scales do not have as good psychometric properties as the Maladaptive style scale [21]. Therefore, we did not use the three defense style scales for the path analysis and logistic regressions models, but only for MANOVAs. Finally, due to the scant presence of engaged and disengaged Studyholics in the current sample, it has not been possible to compare them to detect eventual differences in the use of defense mechanisms. The prevalence of the two types of Studyholic did not allow us to use either parametric or non-parametric analyses.

Despite these limitations, the present study has the merit of having analyzed the defense style of two forms of HSI with the greatest detail possible. To the best of the authors’ knowledge, this is the first study concerning the analysis of defense mechanisms in Study Engagement and problematic overstudying. Moreover, it provides further evidence on the appropriateness of conceptualizing Studyholism as a clinical condition and as an OCD-related disorder (or internalizing rather than externalizing disorder). Additionally, by highlighting that Study Engagement, even if generally characterized by an adaptive defense style, also has some maladaptive defenses, it further supports the need to screen engaged students for relevant clinical disorders that might be hidden by their overstudying behavior.

## 5. Conclusions

The present study analyzed the defense profile of Studyholism and Study Engagement. Among the main findings, we found that defense mechanisms play an important role in predicting Studyholism and Study Engagement and that, generally, while Studyholism is associated with a more maladaptive defense style, Study Engagement is associated with a more adaptive defense style. More specifically, the strongest (and positive) predictor of Studyholism is regression (maladaptive defense), while for Study Engagement it is task-orientation (adaptive defense). Hence, support has been provided to the definition of Studyholism as a new *potential* clinical condition [23,24]. Additionally, through a critical analysis of all the defense mechanisms that proved to be statistically significant predictors of Studyholism, it has been shown that the OCD-related framework is adequate for the conceptualization of Studyholism, in line with previous studies (e.g., [7,12,15]). Moreover, we found that omnipotence and sublimation are negative predictors of Studyholism (in contrast with SUD, which is characterized by higher levels of omnipotence and sublimation [38,39]). Additionally, the results regarding lower sublimation and higher regression are in line with OCD studies [20,29] and, regarding lower sublimation only, with IAD studies [43,44]. Therefore, even if the literature concerning the defense styles of OCD, SUD, and IAD is inconsistent—and, therefore, it is not possible to use these data for definitive conclusions concerning the internalizing and/or externalizing nature of problematic overstudying—we suggest that Studyholism has a defense style that is more similar to OCD than to SUD, hence supporting its conceptualization as an OCD-related disorder. In addition, as IAD is more similar to Studyholism than SUD concerning sublimation, we also recommend taking into account OCD features in IAD. In fact, IAD is not formally recognized as a behavioral addiction [16], and it is critical to avoid a confirmatory approach based on addiction for analyzing excessive behaviors [50]. Finally, regarding Study Engagement, the finding that projection is a positive predictor supports Loscalzo and Giannini’s [7] suggestion that Study Engagement might constitute a coping strategy with paranoid (and anxiety) symptoms. Hence, in line with previous literature (e.g., [7,12,15]), we strongly suggest screening engaged students for adverse outcomes (such as social impairment) and for clinically relevant symptoms that might be hidden by hard studying (such as social anxiety, anxiety, and paranoid ideation). In conclusion, regarding clinical implications, based on the findings highlighting that defense mechanisms play a critical role in both Studyholism and Study Engagement, we suggest that psychodynamic therapies (such as Freudian, Jungian, or sandplay therapies) might be effective in reducing Studyholism, but also in reducing the adverse effects that could be associated with Study Engagement in some youths.

## Figures and Tables

**Table 1:** Descriptive statistics of the variables (*n* = 422).

Variable	*n* Item	Range	M (DS)	Skewness	Kurtosis
Studyholism	4	4–20	12.76 (3.64)	−0.18	−0.53
Study Engagement	4	4–20	14.80 (3.10)	−0.52	0.23
Acting-Out	5	5–43	23.37 (7.30)	0.11	−0.30
Affiliation	2	2–18	9.64 (3.53)	−0.22	−0.33
Undoing	3	3–24	11.04 (4.32)	0.22	−0.35
Anticipation	2	2–18	11.83 (3.17)	−0.38	0.11
Passive Aggressive	5	5–35	19.85 (5.75)	0.002	−0.29
Consumption	3	3–27	10.81 (5.19)	0.58	−0.27
Denial	3	3–20	8.16 (3.57)	0.54	−0.18
Fantasy	1	1–9	5.80 (2.27)	−0.50	−0.58
Reaction Formation	5	5–45	19.98 (6.66)	0.19	−0.01
Primitive Idealization	2	2–18	9.28 (3.70)	0.01	−0.45
Projective Identification	1	1–9	2.08 (1.53)	1.67	2.63
Inhibition	5	5–40	20.65 (7.52)	0.08	−0.52
Isolation of Affect	4	4–33	15.66 (6.31)	0.20	−0.57
Help-Rejecting Complaining	3	3–24	9.65 (4.56)	0.51	−0.28
Omnipotence	6	6–47	22.33 (8.05)	0.24	−0.34
Task-Orientation	2	2–18	12.41 (3.11)	−0.44	0.04
Projection	9	9–64	23.52 (7.92)	1.10	2.37
Pseudo-Altruism	1	1–9	6.51 (1.62)	−0.60	0.05
Regression	2	2–18	8.81 (3.50)	0.01	−0.55
Suppression	2	2–17	9.70 (3.18)	−0.15	−0.10
Withdrawal	3	3–27	17.96 (4.87)	−0.48	−0.01
Splitting	3	3–26	11.87 (4.84)	0.27	−0.41
Somatization	2	2–18	8.04 (3.71)	0.41	−0.40
Sublimation	1	1–9	3.62 (2.42)	0.64	−0.78
Humor	3	5–27	17.59 (4.21)	−0.32	−0.29
Lie (Control) scale	10	31–85	58.62 (9.36)	−0.22	−0.08
Maladaptive style	37	55–229	140.93 (30.04)	0.10	0.08
Image-Distorting style	16	19–106	58.00 (15.56)	0.09	−0.44
Adaptive style	13	37–104	70.94 (10.59)	0.03	0.25

**Table 2:** Zero-order correlations of the study variables (*n* = 422).

Variable	Studyholism	Study Engagement
Acting-Out	0.26 ***	−0.24 ***
Affiliation	0.05	0.05
Undoing	0.14 **	−0.05
Anticipation	0.12 **	0.20 ***
Passive Aggressive	0.17 ***	−0.20 ***
Consumption	0.11 *	−0.13 **
Denial	0.01	−0.16 ***
Fantasy	0.18 ***	−0.15 **
Reaction Formation	0.06	−0.09
Primitive Idealization	0.12 **	−0.05
Projective Identification	0.22 ***	−0.27 ***
Inhibition	0.22 ***	−0.04
Isolation of Affect	0.01	−0.16 ***
Help-Rejecting Complaining	0.26 ***	−0.18 ***
Omnipotence	−0.10 *	−0.09
Task-Orientation	0.13 **	0.35 ***
Projection	0.25 ***	−0.10 *
Pseudo-Altruism	0.15 **	0.03
Regression	0.52 ***	−0.04
Suppression	−0.18 ***	−0.04
Withdrawal	0.40 ***	0.07
Splitting	0.18 ***	−0.001
Somatization	0.41 ***	−0.03
Sublimation	−0.04	−0.03
Humor	−0.09	−0.08
Lie (Control) scale	0.24 ***	−0.11 *
Maladaptive style	0.45 ***	−0.20 ***
Image-Distorting style	0.02	−0.15 **
Adaptive style	0.04	0.10 *

Note. *** *p* ≤ 0.001; ** *p* ≤ 0.01; * *p* < 0.05.

**Table 3:** Standardized path weights for the statistically significant predictors (*n* = 422).

Dependent Variable	Predictor	*β*	*p*
Studyholism	Affiliation	−0.08	0.050
	Anticipation	0.09	0.034
	Projective Identification	0.11	0.015
	Help-Rejecting Complaining	0.11	0.017
	Omnipotence	−0.11	0.016
	Task-Orientation	0.14	<0.001
	Pseudo-Altruism	0.12	0.003
	Regression	0.29	<0.001
	Suppression	−0.12	0.005
	Withdrawal	0.14	0.002
	Somatization	0.17	<0.001
	Sublimation	−0.08	0.045
Study Engagement	Acting-Out	−0.28	<0.001
	Anticipation	0.17	<0.001
	Reaction Formation	−0.13	0.010
	Projective Identification	−0.22	<0.001
	Task-Orientation	0.31	<0.001
	Projection	0.12	<0.001

**Table 4:** Follow-up ANOVAs. DSQ scales by low and high Studyholism (SH) and Study Engagement (SE). Maladaptive style.

Variable		Level	* n *	*M* (*SD*)	* F * ^ § ^	* p *	Partial η^2^
Acting-Out	SH	Low	84	20.34 (7.16)	14.31	<0.001	0.12
		High	24	26.54 (6.78)			
		Total	108	21.72 (7.50)			
	SE	Low	39	27.38 (7.45)	26.81	<0.001	0.23
		High	52	19.17 (7.51)			
		Total	91	22.69 (8.49)			
Undoing	SH	Low	84	9.73 (3.96)	8.31	0.005 ^#^	0.07
		High	24	12.37 (4.01)			
		Total	108	10.31 (4.10)			
	SE	Low	39	11.26 (4.78)	1.23	n.s.	0.01
		High	52	10.06 (5.33)			
		Total	91	10.57 (5.11)			
Passive Aggression	SH	Low	84	18.17 (5.55)	6.45	0.013 ^#^	0.06
		High	24	21.46 (5.77)			
		Total	108	18.90 (5.74)			
	SE	Low	39	22.23 (5.75)	19.21	<0.001	0.18
		High	52	16.90 (5.73)			
		Total	91	19.19 (6.29)			
Consumption	SH	Low	84	9.58 (5.24)	1.96	n.s.	0.02
		High	24	11.25 (4.77)			
		Total	108	9.95 (5.16)			
	SE	Low	39	11.92 (4.88)	6.76	0.011 ^#^	0.07
		High	52	9.25 (4.83)			
		Total	91	10.40 (5.01)			
Fantasy	SH	Low	84	5.20 (2.26)	9.11	0.003 ^#^	0.08
		High	24	6.75 (2.05)			
		Total	108	5.55 (2.30)			
	SE	Low	39	6.33 (2.41)	4.92	0.029 ^#^	0.05
		High	52	5.19 (2.44)			
		Total	91	5.68 (2.48)			
Projective Identification	SH	Low	84	1.62 (1.03)	21.47	<0.001	0.17
		High	24	3.08 (2.18)			
		Total	108	1.94 (1.49)			
	SE	Low	39	3.15 (2.33)	15.89	<0.001	0.15
		High	52	1.65 (1.20)			
		Total	91	2.30 (1.92)			
Help-Rejecting Complaining	SH	Low	84	8.02 (4.00)	15.99	<0.001	0.13
		High	24	12.08 (5.56)			
		Total	108	8.93 (4.68)			
	SE	Low	39	10.36 (4.75)	4.01	0.048 ^#^	0.04
		High	52	8.35 (4.74)			
		Total	91	9.21 (4.82)			
Projection	SH	Low	84	20.38 (5.45)	27.55	<0.001	0.21
		High	24	28.00 (8.62)			
		Total	108	22.07 (7.01)			
	SE	Low	39	24.69 (10.09)	1.78	n.s.	0.02
		High	52	22.15 (8.07)			
		Total	91	23.24 (9.02)			
Regression	SH	Low	84	6.14 (2.95)	74.48	<0.001	0.41
		High	24	11.87 (2.54)			
		Total	108	7.42 (3.73)			
	SE	Low	39	8.79 (3.80)	0.01	n.s.	0.000
		High	52	8.73 (3.68)			
		Total	91	8.76 (3.71)			
Withdrawal	SH	Low	84	14.74 (5.13)	31.16	<0.001	0.23
		High	24	21.25 (4.68)			
		Total	108	16.18 (5.71)			
	SE	Low	39	17.13 (5.89)	1.32	n.s.	0.02
		High	52	18.38 (4.55)			
		Total	91	17.85 (5.18)			
Somatization	SH	Low	84	5.63 (2.42)	68.22	<0.001	0.39
		High	24	10.75 (3.45)			
		Total	108	6.77 (3.42)			
	SE	Low	39	8.44 (4.30)	0.45	n.s.	0.01
		High	52	7.86 (3.80)			
		Total	91	8.11 (4.01)			

Note. DSQ = Defense Style Questionnaire (88 item version); ^§^ = for Studyholism, df = 1,106; for Study Engagement, df = 1,89; ^#^ = not statistically significant using the adjusted alpha level of 0.001.

**Table 5:** Follow-up ANOVAs. DSQ scales by low and high Studyholism (SH) and Study Engagement (SE). Image-Distorting style.

Variable		Level	*n*	*M* (*SD*)	*F* ^§^	*p*	Partial η^2^
Denial	SH	Low	84	7.69 (3.12)	1.32	n.s.	0.01
		High	24	8.58 (4.12)			
		Total	108	7.89 (3.37)			
	SE	Low	39	9.10 (3.31)	5.85	0.018 ^#^	0.06
		High	52	7.29 (3.70)			
		Total	91	8.07 (3.64)			
Isolation of Affect	SH	Low	84	15.92 (6.84)	2.87	n.s.	0.03
		High	24	18.58 (6.69)			
		Total	108	16.51 (6.86)			
	SE	Low	39	18.28 (7.12)	12.68	<0.001	0.13
		High	52	13.35 (6.07)			
		Total	91	15.46 (6.95)			
Omnipotence	SH	Low	84	23.94 (8.05)	1.39	n.s.	0.01
		High	24	21.75 (7.92)			
		Total	108	23.45 (8.04)			
	SE	Low	39	24.54 (7.84)	3.74	n.s.	0.04
		High	52	21.23 (8.24)			
		Total	91	22.65 (8.19)			
Splitting	SH	Low	84	9.79 (4.26)	13.93	<0.001	0.12
		High	24	13.54 (4.64)			
		Total	108	10.62 (4.60)			
	SE	Low	39	11.33 (4.92)	0.06	n.s.	0.001
		High	52	11.60 (4.90)			
		Total	91	11.48 (4.88)			

Note. DSQ = Defense Style Questionnaire (88 item version); ^§^ = for Studyholism, df = 1,106; for Study Engagement, df = 1,89; ^#^ = not statistically significant using the adjusted alpha level of 0.001.

**Table 6:** Follow-up ANOVAs. DSQ scales by low and high Studyholism (SH) and Study Engagement (SE). Adaptive style.

Variable		Level	*n*	*M* (*SD*)	*F* ^§^	*p*	Partial η^2^
Anticipation	SH	Low	84	11.58 (3.34)	8.79	0.004 ^#^	0.08
		High	24	13.79 (2.75)			
		Total	108	12.07 (3.33)			
	SE	Low	39	10.72 (4.4.68)	4.60	0.035 ^#^	0.05
		High	52	12.50 (3.24)			
		Total	91	11.74 (4.00)			
Primitive Idealization	SH	Low	84	8.17 (3.30)	1.53	n.s.	0.01
		High	24	9.17 (4.15)			
		Total	108	8.39 (3.51)			
	SE	Low	39	9.46 (3.98)	0.31	n.s.	0.003
		High	52	8.96 (4.48)			
		Total	91	9.18 (4.26)			
Task-Orientation	SH	Low	84	11.73 (3.06)	0.83	n.s.	0.01
		High	24	12.42 (3.94)			
		Total	108	11.88 (3.27)			
	SE	Low	39	10.97 (3.12)	20.87	<0.001	0.19
		High	52	14.13 (3.37)			
		Total	91	12.78 (3.61)			
Pseudo-Altruism	SH	Low	84	6.30 (1.59)	7.48	0.007 ^#^	0.07
		High	24	7.25 (1.15)			
		Total	108	6.51 (1.55)			
	SE	Low	39	6.36 (1.93)	0.30	n.s.	0.003
		High	52	6.58 (1.83)			
		Total	91	6.48 (1.86)			
Suppression	SH	Low	84	10.59 (2.93)	3.29	n.s.	0.03
		High	24	9.25 (4.05)			
		Total	108	10.30 (3.24)			
	SE	Low	39	9.61 (3.27)	0.04	n.s.	0.000
		High	52	9.48 (3.27)			
		Total	91	9.54 (3.26)			
Sublimation	SH	Low	84	3.58 (2.57)	0.000	n.s.	0.000
		High	24	3.58 (2.57)			
		Total	108	3.58 (2.55)			
	SE	Low	39	3.51 (2.27)	0.01	n.s.	0.000
		High	52	3.46 (2.75)			
		Total	91	3.48 (2.54)			
Humor	SH	Low	84	18.06 (4.08)	4.98	0.028 ^#^	0.05
		High	24	15.79 (5.36)			
		Total	108	17.56 (4.47)			
	SE	Low	39	18.72 (4.97)	1.86	n.s.	0.02
		High	52	17.29 (4.93)			
		Total	91	17.90 (4.97)			

Note. DSQ = Defense Style Questionnaire (88 item version); ^§^ = for Studyholism, df = 1,106; for Study Engagement, df = 1,89; ^#^ = not statistically significant using the adjusted alpha level of 0.001.

**Table 7:** Follow-up ANOVAs. DSQ scales by low and high Studyholism (SH) and Study Engagement (SE). Lie scale and other defenses.

Variable		Level	*n*	*M* (*SD*)	*F* ^§^	*p*	Partial η^2^
Affiliation	SH	Low	84	9.32 (3.86)	0.02	n.s.	0.000
		High	24	9.21 (3.61)			
		Total	108	9.30 (3.79)			
	SE	Low	39	8.97 (4.16)	1.17	n.s.	0.01
		High	52	9.94 (4.27)			
		Total	91	9.53 (4.22)			
Reaction Formation	SH	Low	84	19.29 (7.24)	0.07	n.s.	0.001
		High	24	19.71 (5.78)			
		Total	108	19.38 (6.92)			
	SE	Low	39	19.33 (7.62)	0.97	n.s.	0.01
		High	52	17.85 (6.73)			
		Total	91	18.48 (7.12)			
Inhibition	SH	Low	84	17.93 (7.50)	5.21	0.024 ^#^	0.05
		High	24	22.04 (8.73)			
		Total	108	18.84 (7.94)			
	SE	Low	39	20.54 (8.35)	0.10	n.s.	0.001
		High	52	20.02 (7.60)			
		Total	91	20.24 (7.89)			
		High	52	17.29 (4.93)			
		Total	91	17.90 (4.97)			
Lie (Control) scale	SH	Low	84	55.09 (8.20)	12.89	<0.001	0.11
		High	24	62.17 (9.54)			
		Total	108	56.67 (8.97)			
	SE	Low	39	60.82 (9.93)	6.28	0.014 ^#^	0.07
		High	52	55.71 (9.38)			
		Total	91	57.90 (9.90)			

Note. DSQ = Defense Style Questionnaire (88 item version); ^§^ = for Studyholism, df = 1,106; for Study Engagement, df = 1,89; ^#^ = not statistically significant using the adjusted alpha level of 0.001.

**Table 8:** Follow-up ANOVAs. Defense style scales by low and high Studyholism (SH) and Study Engagement (SE).

Variable		Level	*n*	*M* (*SD*)	*F* ^§^	*p*	Partial η^2^
Maladaptive style	SH	Low	85	119.72 (24.39)	63.26	<0.001	0.37
		High	24	165.42 (26.51)			
		Total	109	129.78 (31.21)			
	SE	Low	39	151.69 (33.30)	11.76	<0.001	0.12
		High	52	127.71 (32.80)			
		Total	91	137.99 (34.93)			
Image-Distorting style	SH	Low	85	57.07 (14.36)	2.52	n.s.	0.02
		High	24	62.46 (15.87)			
		Total	109	58.26 (14.80)			
	SE	Low	39	63.26 (16.41)	8.19	0.005 ^#^	0.08
		High	52	53.46 (15.96)			
		Total	91	57.66 (16.79)			
Adaptive style	SH	Low	85	69.80 (10.75)	0.31	n.s.	0.003
		High	24	71.25 (12.84)			
		Total	109	70.12 (11.20)			
	SE	Low	39	69.36 (14.09)	1.33	n.s.	0.02
		High	52	72.40 (11.08)			
		Total	91	71.10 (12.48)			

Note. ^§^ = for Studyholism, df = 1,107; for Study Engagement, df = 1,89; ^#^ = not statistically significant using the adjusted alpha level of 0.001.

## Data Availability

The dataset is available, upon reasonable request, and for research purposes only, by writing to the corresponding author.
